# Perspectives of Infectious Disease Physicians on *Bartonella quintana* Cases, United States, 2014–2024

**DOI:** 10.3201/eid3012.240655

**Published:** 2024-12

**Authors:** Souci Louis, Grace Marx, Alison F. Hinckley, Shannan N. Rich, Susan E. Beekmann, Philip M. Polgreen, Matthew Kuehnert, Jessica N. Ricaldi, Scott Santibañez

**Affiliations:** Centers for Disease Control and Prevention, Atlanta, Georgia, USA (S. Louis, G. Marx, A.F. Hinckley, S.N. Rich, M. Kuehnert, J.N. Ricaldi, S. Santibañez); University of Iowa Carver College of Medicine, Iowa City, Iowa, USA (S.E. Beekmann, P.M. Polgreen)

**Keywords:** *Bartonella quintana*, homelessness, body louse, body lice, pediculosis, healthcare access, diagnostic testing, surveys, questionnaires, communicable diseases, bacteria, parasites, vector-borne infections, zoonoses, United States

## Abstract

In a US survey of infectious disease specialists, 61 respondents reported seeing >1 *Bartonella quintana* infection during 2014–2024. Diagnostic challenges included limited healthcare provider awareness, inadequate testing, and inconsistent healthcare access among affected populations. Early recognition of *B. quintana* infections is needed to improve outcomes among affected populations.

*Bartonella quintana* is a pathogenic bacterium carried and transmitted to humans by the body louse, *Pediculus humanus humanus*. Clinical manifestations of disease are relapsing fever, bacillary angiomatosis, chronic bacteremia, and endocarditis (*1*). *B. quintana* infections are not nationally notifiable and little is known regarding their incidence and geographic distribution. Barriers to healthcare access in affected human populations and inherent diagnostic challenges might both contribute to underdiagnosis of cases (*2*). However, recent cases have been reported among persons experiencing homelessness (PEH) in New York, New York, and Denver, Colorado, USA (*1*,*3*). 

The Infectious Diseases Society of America’s Emerging Infections Network (EIN) is a healthcare provider–based sentinel network that has >2,800 infectious disease specialists throughout North America (*4*). We evaluated provider-diagnosed *B. quintana* infections reported by EIN members to identify opportunities for improving disease awareness and patient diagnosis.

We sent a 6-question survey to EIN members to collect data regarding *B. quintana* cases, affected populations, and diagnostic challenges (Appendix). We distributed the questionnaire through an electronic mailing list on January 18, 2024, and sent reminder emails on January 25 and February 7. A total of 240 members from 41 US states and the District of Columbia responded; 61 (25%) respondents from 24 states and the District of Columbia stated that they had seen >1 case of *B. quintana* infection within the previous 10 years (Table), and 47 (20%) noted that cases occurred primarily in PEH communities. Other, nonmutually exclusive affected populations included persons with substance use disorders, mental health disorders, HIV infection, and refugee or rural indigenous populations. The most frequently reported obstacles to earlier diagnosis of *B. quintana* infection were the lack of clinical suspicion (88%), knowledge about diagnostic tests (73%), and access to *B. quintana*-specific diagnostic tests (51%). Other challenges included long laboratory turnaround times and inconsistent access to healthcare among affected populations. Free-text responses indicated the value of general clinical knowledge about *B. quintana* infection. For example, one respondent commented, “I have a strong clinical suspicion that there is an association with other endovascular infections that we sometimes miss clinically. For example, a hemorrhagic stroke in someone with a history of homelessness should raise suspicion of this infection. Additionally, any mycotic aneurysm, particularly of the thoracic or abdominal aorta, should raise suspicion for *B. quintana* infection.”

**Table:** Quantitative summary of responses from Emerging Infections Network members across the United States who reported seeing *Bartonella quintana* infection cases during 2014–2024*

Questions	Responses, no. (%)
Question 1. Have you seen any cases?	
a. Recently, 2019–2024, n = 240	
Yes	44 (18)
No	191 (80)
Not sure	5 (2)
b. More remotely, 2014–2018, n = 240	
Yes	34 (14)
No	174 (73)
Not sure	20 (8)
NA (not in practice)	12 (5)
Question 2a. Have you noticed cases within any of the following communities (persons experiencing homelessness, persons with substance use disorder, persons with mental health disorders)? n = 240	
Yes	47 (20)
No	17 (7)
Not sure	5 (2)
NA (no cases)	171 (71)
Question 2b. If yes, in which communities? n = 46 [select any that apply; numbers add up to >100%]	
Persons experiencing homelessness	42 (91)
Persons with substance use disorders	30 (65)
Persons with mental health disorders	29 (63)
Question 3. What do you see as obstacles(s) to earlier diagnosis for patients with *B. quintana* infections? n = 203 [select any that apply; numbers add up to >100%]	
Lack of clinical suspicion by providers	179 (88)
Lack of provider knowledge about optimal diagnostic tests for *B. quintana*	148 (73)
Lack of clinically available *B. quintana*–specific diagnostic tests	103 (51)
No obstacles selected	37 (18)

*Responses were collected from a questionnaire sent to Emerging Infections Network members in 2024 (Appendix). Free-text questions and responses are not shown. NA, not applicable.

We believe that EIN members are seeing *B. quintana* cases in diverse geographic locations across the United States, including in the Southeast (Figure), where *B. quintana* has not been described in the literature (*2*,*5*). We also believe that those findings highlight the importance of increasing clinician awareness of possible *B. quintana* infections among patients at risk for body louse infestation across the United States.

**Figure:**
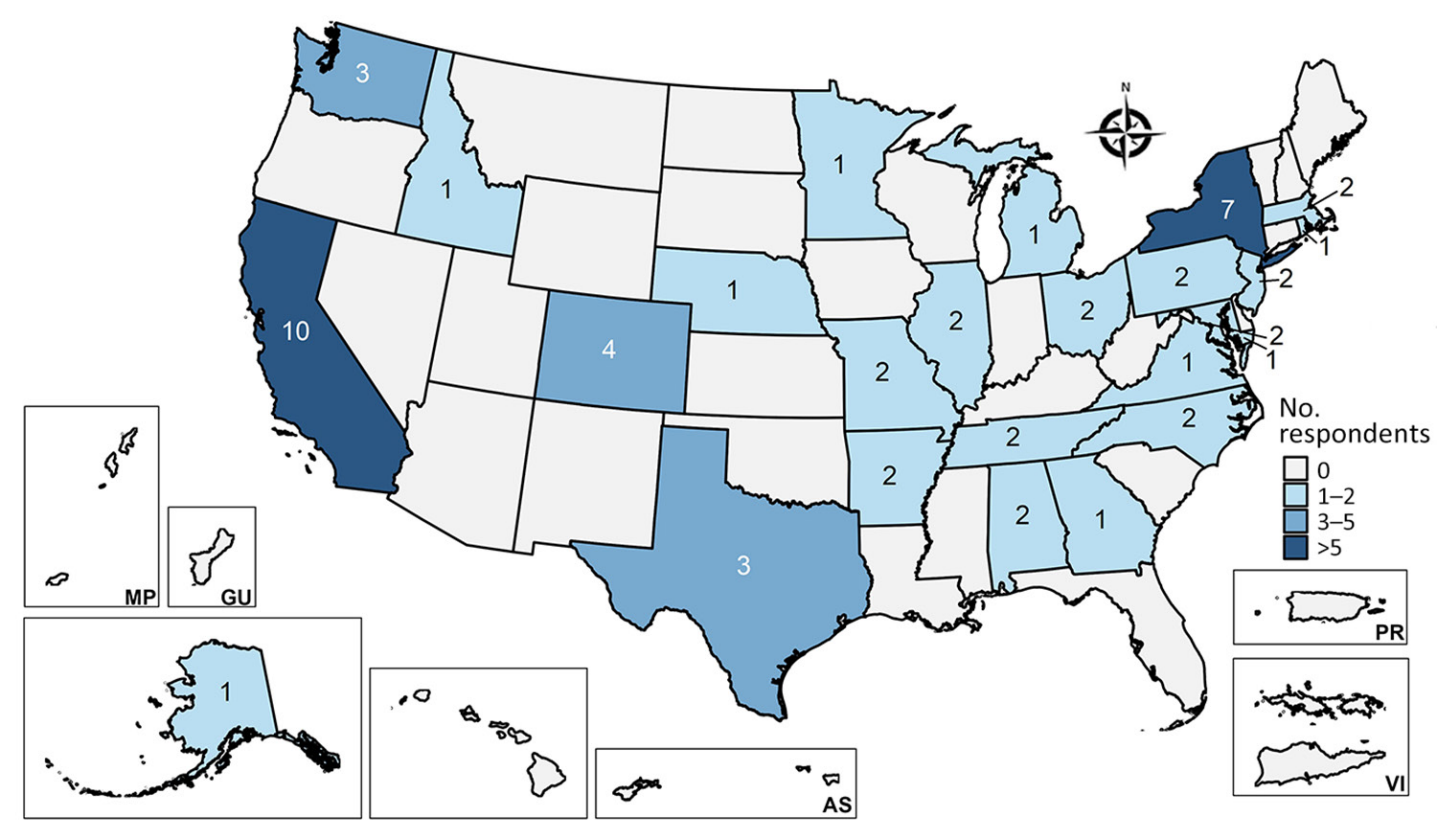
Map indicating numbers and locations of infectious disease physicians responding to survey regarding *Bartonella* *quintana* infection cases, United States, 2014–2024. US states and territories are indicated. The survey was sent to members of The Infectious Diseases Society of America’s Emerging Infections Network in 2024. AS, American Samoa; G, Guam; MP, Northern Marianis Islands; PR, Puerto Rico; VI, Virgin Islands.

Diagnosis of a *B. quintana* infection is challenging because of serologic cross-reactivity with other *Bartonella* spp. and the specific conditions required for a bacterial culture. Several studies suggest that laboratory confirmation could be improved by using molecular testing for detection instead of serologic and culture methods (*6*,*7*). Intentional collaboration between healthcare providers and clinical microbiology laboratories could result in earlier diagnosis and improved treatment outcomes, especially the use of reflexive *B. quintana* molecular diagnostic assays for PEH seeking care for fever of unknown etiology in emergency departments (*2*).

PEH are disproportionately affected by *B. quintana* infections, although several other communities are impacted in the United States (*1*–*3*). Co-existing medical conditions (e.g., behavioral health conditions) and socioeconomic barriers beyond housing instability, such as lack of medical insurance, can further complicate clinical management (*1*). Inconsistent access to running water, showers, and laundry facilities with hot water increases the risk for body lice infestation*.* Limited access to healthcare increases the risk for undiagnosed and untreated *B. quintana* infections that can lead to severe disease. Recognizing complex social determinants of health provides an opportunity to improve prevention, detection, and treatment of *B. quintana* infections.

The first limitation of our study is that, although querying EIN members is an efficient and convenient method to hear from infectious disease specialists, those members are not representative of all healthcare providers in the United States. Second, the EIN members who did respond might not have recalled all of their *B. quintana* cases. Also, reported case locations might have differed from the providers' current practice location.

In conclusion, we consider it critical to increase awareness of *B. quintana* infection risk among certain patient populations, such as PEH, across the United States and increase awareness of diagnostic testing that would most effectively detect active *B. quintana* infections. Promoting early recognition and diagnosis of *B. quintana* infections could result in earlier treatment and improve health outcomes among affected populations.

Appendix Additional information for perspectives of infectious disease physicians on *Bartonella quintana* cases, United States, 2014–2024.
