# Supplementary material for: Perspectives of Infectious Disease Physicians on Bartonella quintana Cases, United States, 2014–2024
Source: Emerg Infect Dis. 2024 Dec;30(12):2702–4. doi: 10.3201/eid3012.240655 (PMC11616639; doi:10.3201/eid3012.240655)
Supplement: Appendix — Additional information for perspectives of infectious disease physicians on Bartonella quintana cases, United States, 2014–2024. [file 24-0655-Techapp-s1.pdf]

Article DOI: https://doi.org/10.3201/eid3012.240655

EID cannot ensure accessibility for supplementary materials supplied by authors. Readers who have difficulty accessing supplementary content should contact the authors for assistance.

Perspectives of Infectious Disease Physicians on *Bartonella quintana* Cases, United States, 2014–2024

Appendix

Survey Distribution and Questions

EIN listserv subscribers were US infectious disease physicians within the Emerging Infections Network, other infectious disease healthcare professionals who had advanced degrees (e.g., PharmD, nurse practitioner, and physician assistant), public health members, and international members. A listserv email that included a link to the electronic survey was sent on January 18, 2024. Completion of the survey was voluntary. Listserv subscribers received 2 email reminders (January 25 and February 7, 2024) before the survey closed on February 14, 2024.

Emerging Infections Network questionnaire

Bartonella quintana Infections

1. Have you seen any cases...

a. ...recently (2019-present)?

_ Yes

_ No

_ Not sure

b. ...more remotely (2014–2018)?

_ Yes

_ No

_ Not sure

_ Not applicable (was not in practice)

2. Have you noticed cases within any of the following communities (persons experiencing homelessness, persons with substance use disorder, persons with mental health disorders)?

a. _ Yes [select all that apply below]

_ No

_ Not sure

_ Not applicable (no cases)

b. If yes, in which communities?

_ People experiencing homelessness

_ People with substance use disorders

_ People with mental health disorders

_ Other, *please specify*:

3. What do you see as obstacle(s) to earlier diagnosis for patients with *B. quintana* infections?

_ Lack of clinical suspicion by providers

_ Lack of provider knowledge about optimal diagnostic tests for *B. quintana*

_ Lack of clinically available *B. quintana*-specific diagnostic tests

_ Other, *please specify*:

4. Please select the state of your clinical practice: *[Drop down menu with US states]*

5. If you have had a prior case in the past 10 years and would be interested in contributing to a future case series, please enter your:

Name: *[Open text field]* and email: *[Open text field]*

6. Any comments about *B. quintana* are welcome:

[Open text field]

If you recover a *B. quintana* isolate and would like to submit to the CDC reference repository, please refer to the Bartonella Special Study test order on the CDC test directory for contact information.
